# Supplementary material for: Investigating Neolithic caprine husbandry in the Central Pyrenees: Insights from a multi-proxy study at Els Trocs cave (Bisaurri, Spain)
Source: PLoS One. 2021 Jan 6;16(1):e0244139. doi: 10.1371/journal.pone.0244139 (PMC7787385; doi:10.1371/journal.pone.0244139)
Supplement: S1 Table — A) Radiocarbon dates from Els Trocs cave with lab references numbers (Mams = Mannheim AMS facility at the Curt-Engelhorn-Centre for Achaeometry; Beta = Beta Analytic), stratigraphic context, materials dated, and available isotopic data. Calibration is with OxCal v.4.4.2 (https://c14.arch.ox.ac.uk (1)) using IntCal20 atmospheric curve (2). B) Results of the Bayesian model (Phase sequential analysis) for the radiocarbon dates from Els Trocs cave. Calibration and modelled is with OxCal v.4.4.2 (https://c14.arch.ox.ac.uk (1)) using IntCal20 atmospheric curve (2). (DOCX) [file pone.0244139.s002.docx]

**S1 Table. A) Radiocarbon dates from Els Trocs cave with lab references numbers (Mams= Mannheim AMS facility at the Curt-Engelhorn-Centre for Achaeometry; Beta= Beta Analytic), stratigraphic context, materials dated, and available isotopic data. Calibration is with OxCal v.4.4.2 (**[**https://c14.arch.ox.ac.uk**](https://c14.arch.ox.ac.uk) (1)**) using IntCal20 atmospheric curve(2).**

| **PHASE** | **LAB NUMBER** | **CONTEXT** | **MATERIAL** | **14C yr BP** | **UNMODELLED CAL. yr BCE 2σ (95.4%)** | **δ13C**  **(‰)** | **C:N** | **%C** | **REFERENCE** |
| --- | --- | --- | --- | --- | --- | --- | --- | --- | --- |
| **TROCS I** | Mams – 16163 | SU 105 | Human bone | 6285±25 | 5312-5213 | -23.0 | 3.3 | 47.0 | (3) |
|  | Mams – 16159 | SU 63 | Human bone | 6280±25 | 5312-5212 | -21.5 | 3.2 | 43.3 | (3) |
|  | Mams – 16164 | SU 101 | Human bone | 6249±25 | 5308-5073 | -19.8 | 3.2 | 38.9 | (3) |
|  | Mams – 16168 | SU 20 | Human bone | 6249±28 | 5309-5073 | -28.5 | 3.2 | 32.0 | (3) |
|  | Mams – 16166 | SU 20 | Human bone | 6234±28 | 5304-5065 | -27.5 | 3.3 | 42.3 | (3) |
|  | Mams – 16162 | SU 85 | Human bone | 6218±24 | 5298-5059 | -16.5 | 3.2 | 40.5 | (3) |
|  | Mams – 16161 | SU 82 | Human bone | 6217±25 | 5298-5057 | -16.0 | 3.2 | 32.4 | (3) |
|  | Beta – 316512 | SU 53 | Crop *(Triticum aestivum-durum*) | 6080±40 | 5206-4846 | -22.6 | - | - | (3) |
|  | Beta – 284150 | SU 20 | Crop *(Triticum aestivum-durum*) | 6070±40 | 5205-4843 | -22.8 | - | - | (3) |
|  | Beta – 295782 | SU 63 | Animal bone (*Ovis*) | 6060±40 | 5203-4841 | -20.1 | - | - | (3) |
|  | Beta – 316514 | SU 16 | Crop *(Triticum aestivum-durum*) | 6050±40 | 5201-4837 | -23.7 | - | - | (3) |
|  | Beta – 438433 | SU 20 | Animal bone (*Ovis*) | 6010±30 | 4994-4800 | -20.5 | - | - | Unpublished |
| **TROCS II** | Beta - 316511 | SU 14 | Crop *(Triticum aestivum-durum*) | 5590±40 | 4498-4347 | -24.7 | - | - | (3) |
|  | Beta - 316515 | SU 8 | Crop *(Triticum aestivum-durum*) | 5590±40 | 4498-4347 | -24.1 | - | - | (3) |
|  | Beta - 319513 | SU 77 | Crop *(Triticum aestivum-durum*) | 5580±40 | 4493-4345 | -24.3 | - | - | (3) |
| **TROCS III** | Mams - 16165 | SU 1 | Human bone | 5035±23 | 3949-3715 | -20.5 | 3.2 | 36.1 | (3) |
|  | Mams - 16160 | SU 69 | Human bone | 5008±23 | 3941-3660 | -17.5 | 3.2 | 44.6 | (3) |
|  | Mams - 14856 | SU 38 | Human bone | 5005±27 | 3943-3657 | -20.4 | 3.2 | 40.6 | (3) |
|  | Mams - 16167 | SU 6 | Human bone | 4512±25 | 3354-3101 | -22.8 | 3.2 | 41.8 | (3) |
|  | Beta - 316510 | SU 1 | Crop *(Triticum aestivum-durum*) | 4410±40 | 3327-2912 | -23.2 | - | - | (3) |

**B) Results of the Bayesian model (Phase sequential analysis) for the radiocarbon dates from Els Trocs cave. Calibration and modelled is with OxCal v.4.4.2 (**[**https://c14.arch.ox.ac.uk**](https://c14.arch.ox.ac.uk) **(1)) using IntCal20 atmospheric curve(2).**

| **ELS TROCS CAVE/NEOLITHIC PHASES**  **Amodel = 97.1/Aoverall = 95.8** | | | | | |
| --- | --- | --- | --- | --- | --- |
|  | | **14C yr BP** | **MODELLED CAL. yr BCE** | | **A (individual agreement indices)** |
|  |  |  | **1σ (68.2%)** | **2σ (95.4%)** |  |
| **TROCS III** | **End Trocs III** |  | 3261-2930 | 3322-2588 |  |
|  | **Span Trocs III (yr)** |  | 586-868 | 493-958 |  |
|  | Beta – 316510 | 4410±40 | 3328-3019 | 3338-2926 | 72.5 |
|  | Mams – 16167 | 4512±25 | 3351-3132 | 3356-3105 | 96.4 |
|  | Mams – 14856 | 5005±27 | 3895-3711 | 3941-3657 | 101.4 |
|  | Mams – 16160 | 5008±23 | 3897-3731 | 3939-3660 | 101.1 |
|  | Mams – 16165 | 5035±23 | 3935-3778 | 3946-3714 | 96.3 |
|  | **Start Trocs III** |  | 4138-3877 | 4303-3793 |  |
| **TROCS II** | **End Trocs II** |  | 4421-4318 | 4451-4152 |  |
|  | **Span Trocs II (yr)** |  | 0-51 | 0-104 |  |
|  | Beta – 319513 | 5580±40 | 4444-4371 | 4486-4349 | 106.7 |
|  | Beta – 316515 | 5590±40 | 4446-4370 | 4488-4351 | 108.4 |
|  | Beta – 316511 | 5590±40 | 4446-4370 | 4488-4351 | 108.3 |
|  | **Start Trocs II** |  | 4509-4388 | 4693-4362 |  |
| **TROCS I** | **End Trocs I** |  | 4938-4834 | 4978-4752 |  |
|  | **Span Trocs I (yr)** |  | 323-411 | 289-455 |  |
|  | Beta – 438433 | 6010±30 | 4990-4895 | 5000-4846 | 91.6 |
|  | Beta – 316514 | 6050±40 | 5006-4907 | 5200-4850 | 104.5 |
|  | Beta – 295782 | 6060±40 | 5031-4930 | 5203-4850 | 104.9 |
|  | Beta – 284150 | 6070±40 | 5039-4936 | 5206-4850 | 104.8 |
|  | Beta – 316512 | 6080±40 | 5046-4939 | 5207-4853 | 104.1 |
|  | Mams – 16161 | 6217±25 | 5218-5073 | 5297-5056 | 99.8 |
|  | Mams – 16162 | 6218±24 | 5218-5073 | 5296-5058 | 99.7 |
|  | Mams – 16166 | 6234±28 | 5295-5079 | 5302-5064 | 97.3 |
|  | Mams – 16168 | 6249±28 | 5301-5132 | 5306-5072 | 95.5 |
|  | Mams – 16164 | 6249±25 | 5301-5208 | 5305-5074 | 94.8 |
|  | Mams – 16159 | 6280±25 | 5296-5215 | 5309-5211 | 99.2 |
|  | Mams – 16163 | 6285±25 | 5274-5216 | 5308-5213 | 100.1 |
|  | **Start Trocs I** |  | 5340-5275 | 5413-5237 |  |

**References**

1. Bronk Ramsey C. Bayesian analysis of radiocarbon dates. Radiocarbon. 2009; 51(1): 337–360.

2. Reimer P, Austin W, Bard E, Bayliss A, Blackwell P, Bronk Ramsey C, et al. The IntCal20 Northern Hemisphere radiocarbon age calibration curve (0–55 cal kBP). Radiocarbon. 2020; 62 (4): 725-757.

3. Rojo Guerra MA, Peña Chocarro L, Royo Guillén JI, Tejedor Rodríguez C, García Martínez De Lagrán I, Arcusa Magallón H, et al. Pastores trashumantes del Neolítico Antiguo en un entorno de alta montaña: secuencia crono-cultural de la Cova de Els Trocs (San Feliú de Veri, Huesca). Boletín del Seminario de Estudios de Arte y Arqueología. 2013;LXXIX:9-55.
