# Supplementary material for: Investigating Neolithic caprine husbandry in the Central Pyrenees: Insights from a multi-proxy study at Els Trocs cave (Bisaurri, Spain)
Source: PLoS One. 2021 Jan 6;16(1):e0244139. doi: 10.1371/journal.pone.0244139 (PMC7787385; doi:10.1371/journal.pone.0244139)
Supplement: S2 Table — Number of faunal remains (NR) in each Neolithic phase. (DOCX) [file pone.0244139.s003.docx]

**S2 Table. Cova de Els Trocs. Number and percentage of vertebrate remains (NR) in each period.**

| **PERIOD** | **CLASS** | **NR** | **%** |
| --- | --- | --- | --- |
| **TROCS I** | Mammal | 6527 | 96 |
|  | [large & medium]^a^ | [5194] | [76] |
|  | [small]^a^ | [1333] | [20] |
|  | Bird | 107 | 2 |
|  | Reptile | 155 | 2 |
|  | Amphibian | 13 | <1 |
|  | *Total* | *6802* | *100* |
| **TROCS II** | Mammal | 6330 | 98 |
|  | [large & medium]^a^ | [5444] | [85] |
|  | [small]^a^ | [886] | [14] |
|  | Bird | 52 | 1 |
|  | Reptile | 39 | 1 |
|  | Amphibian | 3 | <1 |
|  | *Total* | *6424* | *100* |
| **TROCS III** | Mammal | 5636,5 | 97 |
|  | [large & medium]^a^ | [4482,5] | [77] |
|  | [small]^a^ | [1154] | [20] |
|  | Bird | 102 | 2 |
|  | Reptile | 78 | 1 |
|  | Amphibian | 19 | <1 |
|  | *Total* | *5835,5* | *100* |
| **TOTAL** | Mammal | 18493,5 | 97 |
|  | [large & medium]^a^ | [15120,5] | [79] |
|  | [small]^a^ | [3373] | [18] |
|  | Bird | 261 | 1 |
|  | Reptile | 272 | 1 |
|  | Amphibian | 35 | <1 |
|  | *Total* | 19061,5 | 100 |

^a^ The numbers of large, medium and small mammals are shown in brackets. Their total is recorded under Mammal. Large and medium-sized species are those listed in Table 1. The small mammal fraction includes Chiroptera, Rodentia, Insectivora and Carnivora.
