# Supplementary material for: Investigating Neolithic caprine husbandry in the Central Pyrenees: Insights from a multi-proxy study at Els Trocs cave (Bisaurri, Spain)
Source: PLoS One. 2021 Jan 6;16(1):e0244139. doi: 10.1371/journal.pone.0244139 (PMC7787385; doi:10.1371/journal.pone.0244139)
Supplement: S3 Table — Numbers and ratios of sheep (O) and goats (C) and percentages of sheep. The numbers of deciduous lower fourth premolars (dLP4), scapulae (SC), distal humeri (dHU), proximal radii (pRA), distal metacarpals, (MTC), distal metatarsals (MTT), astragali (AST) and calcanea (CAL) are given as x: y, where x = number of sheep teeth or bones and y = number of goat teeth or bones. (DOCX) [file pone.0244139.s004.docx]

**S3 Table. MNE and ratios of sheep (O) and goats (C) and average percentage of sheep in each occupation phase.**

| **PERIOD** |  | **dLP4** | **LP4** | **SC** | **dHU** | **pRA** | **dMTC** | **dMTT** | **AST** | **CAL** | **TOTAL** |
| --- | --- | --- | --- | --- | --- | --- | --- | --- | --- | --- | --- |
|  |  | **O:C** | **O:C** | **O:C** | **O:C** | **O:C** | **O:C** | **O:C** | **O:C** | **O:C** |  |
| **Trocs I** | N | 29:0 | 5:1 | 27:1 | 15:3 | 9:0 | 10:0 | 13:0 | 16:0 | 8:0 | 132:5 |
|  | ratio |  |  |  |  |  |  |  |  |  | 26:1 |
|  | % sheep |  |  |  |  |  |  |  |  |  | 96 |
|  |  |  |  |  |  |  |  |  |  |  |  |
| **Trocs II** | N | 24:1 | 5:1 | 8:2 | 16:1 | 7:1 | 5:2 | 7:0 | 11:0 | 3:0 | 86:8 |
|  | ratio |  |  |  |  |  |  |  |  |  | 11:1 |
|  | % sheep |  |  |  |  |  |  |  |  |  | 91 |
|  |  |  |  |  |  |  |  |  |  |  |  |
| **Trocs III** | N | 10:0 | 8:1 | 20:1 | 8:2 | 6:2 | 6:0 | 10:1 | 14:2 | 7:0 | 89:9 |
|  | ratio |  |  |  |  |  |  |  |  |  | 10:1 |
|  | % sheep |  |  |  |  |  |  |  |  |  | 91 |
|  |  |  |  |  |  |  |  |  |  |  |  |
| **TOTAL** | N | 63:1 | 18:3 | 55:4 | 39:6 | 22:3 | 21:2 | 30:1 | 41:2 | 18:0 | 307:22 |
|  | ratio |  |  |  |  |  |  |  |  |  | 14:1 |
|  | % sheep |  |  |  |  |  |  |  |  |  | 93 |

The numbers of deciduous lower fourth premolars (dLP4), lower fourth premolars (LP4), scapulae (SC), distal humeri (dHU), proximal radii (pRA), distal metacarpals, (MTC), distal metatarsals (MTT), astragali (AST) and calcanea (CAL) are given as x : y, where x = number of sheep teeth or bones and y = number of goat teeth or bones.
