# Supplementary material for: Investigating Neolithic caprine husbandry in the Central Pyrenees: Insights from a multi-proxy study at Els Trocs cave (Bisaurri, Spain)
Source: PLoS One. 2021 Jan 6;16(1):e0244139. doi: 10.1371/journal.pone.0244139 (PMC7787385; doi:10.1371/journal.pone.0244139)
Supplement: S4 Table — (DOCX) [file pone.0244139.s005.docx]

**S4 Table. Caprines MNE and percentages of perinatal elements in each Neolithic phase**

|  | **TROCS I** | | | **TROCS II** | | | **TROCS III** | | |
| --- | --- | --- | --- | --- | --- | --- | --- | --- | --- |
|  | Others | Perinatals | %perinatals | Others | Perinatals | %perinatals | Others | Perinatals | %perinatals |
| **MAX** | 17 | 8 | 32 | 12 | 7 | 37 | 11 | 5 | 31 |
| **MAN** | 27 | 10 | 27 | 21 | 7 | 25 | 21 | 4 | 16 |
| **SC** | 25 | 27 | 52 | 12 | 13 | 52 | 20 | 15 | 43 |
| **HU** | 11 | 18 | 62 | 15 | 12 | 44 | 14 | 10 | 42 |
| **RA** | 17 | 15 | 47 | 19 | 25 | 57 | 26 | 6 | 19 |
| **UL** | 7 | 11 | 61 | 13 | 10 | 43 | 8 | 7 | **47** |
| **MTC** | 13 | 20 | 61 | 8 | 9 | **67** | 9 | 7 | 44 |
| **PEL** | 13 | 14 | 52 | 14 | 7 | 33 | 14 | 3 | 17 |
| **FE** | 12 | 15 | 56 | 9 | 11 | 55 | 8 | 6 | 43 |
| **TI** | 15 | 23 | 60 | 15 | 14 | 48 | 16 | 10 | 38 |
| **AST** | 11 | 10 | 48 | 12 | 2 | 14 | 16 | 3 | 16 |
| **CAL** | 9 | 10 | 53 | 6 | 5 | 45 | 6 | 3 | 33 |
| **MTT** | 8 | 18 | **69** | 8 | 6 | 58 | 10 | 7 | 41 |
| **Ʃ** | 185 | 199 | **52%** | 164 | 128 | **44%** | 179 | 86 | **32%** |
| **Total MNE** | **384** | |  | **292** | |  | **265** | |  |
