# Supplementary material for: Investigating Neolithic caprine husbandry in the Central Pyrenees: Insights from a multi-proxy study at Els Trocs cave (Bisaurri, Spain)
Source: PLoS One. 2021 Jan 6;16(1):e0244139. doi: 10.1371/journal.pone.0244139 (PMC7787385; doi:10.1371/journal.pone.0244139)
Supplement: S8 Table — (DOCX) [file pone.0244139.s009.docx]

| **Eggs** | **Couple of phases** | ***P **** |
| --- | --- | --- |
| All helminths | Trocs III *vs.* Trocs I | 0.0293 |
|  | Trocs III *vs.* Trocs II | NS ** |
|  | Trocs II *vs.* Trocs I | NS |
|  | Trocs III *vs.* Trocs I+II | 0.0354 |
| *Ascaris* sp. | Trocs III *vs.* Trocs I | NS |
|  | Trocs III *vs.* Trocs II | NS |
|  | Trocs II *vs.* Trocs I | NS |
|  | Trocs III *vs.* Trocs I+II | NS |
| *Capillaria* sp. | Trocs III *vs.* Trocs I | 0.0108 |
|  | Trocs III *vs.* Trocs II | NS |
|  | Trocs II *vs.* Trocs I | NS |
|  | Trocs III *vs.* Trocs I+II | 0.0066 |
| *Dicrocoelium* sp. | Trocs III *vs.* Trocs I | 0.0359 |
|  | Trocs III *vs.* Trocs II | NS |
|  | Trocs II *vs.* Trocs 1 | NS |
|  | Trocs III *vs.* Trocs I+II | NS |
| *Fasciola* sp. | Trocs III *vs.* Trocs 1 | NS |
|  | Trocs III *vs.* Trocs II | NS |
|  | Trocs II *vs.* Trocs 1 | NS |
|  | Trocs III *vs.* Trocs I+II | NS |
| *Paramphistomum* sp. | Trocs III *vs.* Trocs I | NS |
|  | Trocs III *vs.* Trocs II | NS |
|  | Trocs II *vs.* Trocs 1 | NS |
|  | Trocs III *vs.* Trocs I+II | NS |
| *Trichuris* sp. | Trocs III *vs.* Trocs 1 | NS |
|  | Trocs III *vs.* Trocs II | NS |
|  | Trocs II *vs.* Trocs 1 | NS |
|  | Trocs III *vs.* Trocs I+II | NS |

**S8 Table Results of the Fisher’s exact test on the helminth eggs samples (all, *Ascaris* sp., *Capillaria* sp., *Dicrocoelium* sp., *Fasciola* sp., *Paramphistomum* sp., and *Trichuris* sp.),** **by occupation phase.**
