# Supplementary material for: Investigating Neolithic caprine husbandry in the Central Pyrenees: Insights from a multi-proxy study at Els Trocs cave (Bisaurri, Spain)
Source: PLoS One. 2021 Jan 6;16(1):e0244139. doi: 10.1371/journal.pone.0244139 (PMC7787385; doi:10.1371/journal.pone.0244139)
Supplement: S1 File — Supplementary material. (DOC) [file pone.0244139.s011.doc]

**S1 File. Material and methods. Supplementary material.**

*Estimation of age-at-death distributions*

The fusion stage of the bones was assigned to the groups reported by Zeder (1), with the exception of group G (based on late fusion or total fusion of proximal humerus). The low numbers of proximally fused humeri were all assigned to group F. The remains of foetal and neonatal individuals were registered as perinatals. Based on morphological and metrical criteria they were counted separately from those of older infants, juveniles and sub-adults, featuring also unfused epiphyses.

The average number of unfused elements for each of the fusion groups is calculated by the following formula:

*(MNE p+u/MNE f+v+u+p)*100*,

where MNE= minimum number of elements, f=fused bones, v=fusing bones, u=unfused bones and p=perinatal bones. The result enables to estimate the rate of survival in each age group. Comparing survivorship curves computed this way in the three Neolithic assemblages provides an overview of the caprines kill-off pattern over the fusion sequence.

*Isotopic analyses*

The eleven sheep mandibles analysed in this work were selected among the total sample of mandibles because they comprise optimal conditions to perform the isotopic analyses. They were positively identified as sheep following diagnostic morphological criteria (43, 46), present second molar (M2) crowns already formed and some also displayed the M3 just developed or in an advanced stage of formation.

Sheep M2 and M3 molars were obtained by performing a small incision in the lingual side of the corpus of each mandible. Then, they were sampled on enamel following a serial or sequential sampling perpendicular to the tooth growth axis, from the apex to the enamel root junction (hereafter ERJ) covering the whole crown height. A binocular magnifying glass with an integrated LED light was used in these processes.

Enamel sampling was performed on the buccal side of the tooth, particularly on the anterior lobe for M2, and the middle lobe for M3. Each sample involves slightly more than one-millimetre-wide horizontal band. Samples are distanced by intervals of 1 to 1.5 mm covering the whole crown height. On M2, a total of 9 to 19 samples were drilled out. On M3, a total of 10 to 19 samples were drilled out. Positions of samples along each tooth crown were noted as the distance (in mm) from ERJ, all of them related to the final development stage of tooth crown.

Samples weighing 3.5 to 7.5 mg were chemically treated following published proposals in Tornero et al. (2), and ensuring elimination of exogenous carbonates. Samples were placed for four hours in 0.1 M acetic acid (0.1 ml solution/mg of sample) and rinsed in distilled water and dried. During chemical treatment samples lost an average of 35.4 ± 5.64% of their weight.

Analyses of δ^18^O and δ^13^C values were performed at the Service de Spectrométrie de Masse isotopique du Muséum national d'Histoire naturelle (SSMIM) in Paris (France), with technical assistance by D. Fiorillo and scientific supervision from Dr M. Balasse (both UMR7209/MNHN). Dry samples weighting ~600 micrograms were introduced on a Kiel IV device interfaced to a Delta V Advantage isotope ratio mass spectrometer (IRMS). Samples were reacted under vacuum with 100% phosphoric acid at 70ºC in individual vessels and purified in an automated cryogenic distillation system. All samples (n = 251) were measured in 10 different analytical series. Accuracy and precision of the measurements were checked using an internal laboratory calcium carbonate standard (Marbre LM normalized to NBS 19). Over the period of analysis a total of 75 Marbre LM samples gave a mean δ^13^C value of +2.12 ± 0.027 ‰ (1σ) (expected value +2.13 ‰) and δ^18^O value of -1.72 ± 0.120 ‰ (1σ) (expected value -1.83 ‰). Mean analytical precision of Marbre LM within each series was +0.02 ± 0.008 for δ^13^C and +0.04 ± 0.009 for δ^18^O values. δ^13^C and δ^18^O values are here expressed in Vienna-Pee Dee Belemnite (V-PDB) standard. Results obtained were described when necessary using both parametric (Anova, t-Test) and non-parametric (Kruskal-Wallis, Kolmogorov-Smirnov) test, setting significance at a p value <0.05 (with Past program, version 2.15).

Sequential oxygen values used to assess the season and seasonality of birth are normalized following Balasse et al., proposal (3). Oxygen data sets are first modeled following a cosine-function where different key parameters are used to describe the oxygen sequences. Later, modeled sequences were normalized using the ratio of the parameters x_0_ and X. Results of the modelization of oxygen sequences from M2 are presented in S7Table.

We used reference data sets from Blaise and Balasse (4), representing births occurring in late winter (late January to early February) and early autumn (mid September) in the experimental Carmejane farm (CF), South-east France; from Balasse et al. (3), representing birth occurring in mid spring (end of April to beginning of May) in a working farm on the island of Rousay (ROU), in the Orkney Islands, Scotland; and from Tornero et al. (5), representing births occurring in early autumn (early October) from a herd raised in the Ebro Valley, Spain. Details on management, maintenance and environmental context of these breeds can be found on mentioned published studies.

*Palaeoparasitological analyses*

Palaeoparasitological analyses were performed on a total of forty-four sediment samples (S6Table) at the TRACES laboratory CNRS UMR 5608 Toulouse II Jean-Jaurès. They were treated following the RHM method developed by Dufour and Le Bailly (6). Briefly, ten grams of sediment were rehydrated for a week in a specific solution composed by glycerinated water (5%) and trisodium phosphate (0,5%). This solution was then homogenized by crushing and ultrasound (50-60 Hz) processing for one minute. Then the sample was sieved through four increasingly smaller micro-sieves of 300 μm, 160 μm, 50 μm and 25 μm. The remains retained in the 50 μm and 25 μm sieves were placed in polystyrene tubes with formalin solution to avoid the development of moulds. In order to identify the parasite remains ten slides of each fraction were prepared and examined under the microscope (Leica DM-1000 LED) associated with a camera (Leica ICC50-HD.

Statistical calculations were carried out using Epi-Info® (Center for Disease Control, Atlanta, GA).

**References**

1. Zeder MA. Reconciling rates of long bone fusion and tooth eruption and wear in sheep (*Ovis*) and goat (*Capra*). In: Ruscillo D, editor. Recent advances in ageing and sexing animal bones. Oxford: Oxbow Books; 2006. p. 87–118.

2. Tornero C, Balasescu A, Ughetto-Monfrin J, Voinea V, Balasse M. Seasonality and season of birth in early Eneolithic sheep from Cheia (Romania): methodological advances and implications for animal economy. Journal of Archaeological Science. 2013;40:4039-55.

3. Balasse M, Obein G, Ughetto-Monfrin J, Mainland I. Investigating seasonality and season of birth in past herds: a reference set of sheep enamel stable oxygen isotope ratios. Archaeometry. 2012;54(2):349-68.

4. Blaise E, Balasse M. Seasonality and season of birth of modern and late Neolithic sheep from south-eastern France using tooth enamel δ^18^O analysis. Journal of Archaeological Science. 2011;38(11):3085-93.

5. Tornero C, Aguilera M, Ferrio JP, Arcusa H, Moreno-García M, García-Reig S, et al. Vertical sheep mobility along the altitudinal gradient through stable isotope analyses in tooth molar bioapatite, meteoric water and pastures: A reference from the Ebro valley to the Central Pyrenees. Quaternary International. 2018;484 (10):94-106.

6. Dufour B, Le Bailly M. Testing new parasite egg extraction methods in paleoparasitology and an attempt at quantification. International Journal of Paleopathology. 2013;3:199-203.
