# Supplementary material for: Investigating Neolithic caprine husbandry in the Central Pyrenees: Insights from a multi-proxy study at Els Trocs cave (Bisaurri, Spain)
Source: PLoS One. 2021 Jan 6;16(1):e0244139. doi: 10.1371/journal.pone.0244139 (PMC7787385; doi:10.1371/journal.pone.0244139)
Supplement: S2 File — Supplementary material. (DOC) [file pone.0244139.s012.doc]

**S2 File. Main results from our pilot study on sequential sampling molar teeth for isotopic analysis of modern transhumant sheep in Iberia. Supplementary material.**

In 2005 Balasse and Ambrose (1) demonstrated the utility of δ^13^C values to investigate altitudinal movements of modern livestock in Kenya. However, the combination of carbon and oxygen isotope data of sequentially sampled teeth has proved to be a more valid approach to analyse archaeological and modern samples (2-7). Back in 2015, we developed a pilot study with two modern sheep specimens collected from one of the last flocks that still perform vertical mobility from over winter valley locations of the Ebro river basin up to the central Pyrenees in Spain. In that early study, the second lower molars of these individuals, covering a period from April/May 2014 to February/March 2015, provided a negative co-variation of δ^18^O and δ^13^C values along the crown sequences: as animals moved to C3-dominated higher elevations in the summer, low δ^13^C values were paralleled by high δ^18^O values. These results were interpreted according to local rainfall distribution data, seasonal patterns in δ^18^O of sampled meteoric water, and vegetation changes and δ^13^C values in pastures along the altitudinal gradient in the area, providing a reliable analytical framework to explain data obtained from archaeological sheep specimens (3).

In short, monthly δ^18^O values in meteoric water obtained in valley (Zaragoza station) and mountain (Jaca station) positions described the same type of seasonal oscillation observed in the teeth sheep, with enriched values during the warm months and depleted values during the cold months (see Fig.4:7 in Tornero et al (3)). This result demonstrated that sequential δ^18^O measures in bioapatite of tooth enamel were fixing well the seasonal variation of δ^18^O values in meteoric water, independently of the rapid, seasonal, vertical mobility of sheep, and were not affected by other factors (*i.e.*, the altitudinal gradient of δ^18^O values of precipitation, due to a gradual removal of moisture from uplifted air masses, or the potential incorporation of melted water from surface snow with depleted δ^18^O values), eliminating or reversing the natural oscillation of δ^18^O values in tooth molars. Conversely, pastures analyzed along the altitudinal gradient (8, 9) showed a decrease in δ^13^C values with altitude, linked to the seasonal availability of precipitation and vegetation differences among locations. During summer, sheep grazing in the mountain pastures had access to grasses benefitting from an important amount of precipitation. This factor could explain the lower δ^13^C values registered in the sampled sheep during the summer event because plants show lower water use efficiency due to the reduction of stomatal conductance (10). Contrary, during the maximum winter event sheep registered their highest δ^13^C values, in compliance with high δ^13^C values measured in plants collected in the low altitudinal locations of the gradient. In this case, enriched δ^13^C values in sheep sequences linked to overwinter periods would be a consequence not only of the differences in precipitation amounts but also in plant traits between pastures from high mountains and valley locations. Indeed, in that study plant communities from subalpine pastures showed a reduced functional diversity, as compared to the Mediterranean communities (8, 9), and a decrease in δ^13^C, with a dissimilarity in functional diversity of physiological traits in grassland plants (11) in the Mediterranean region (12) and the Ebro Basin area (13).

In sum, this pilot study allowed us the recognition of sheep vertical mobility practices from the Ebro Valley to the Central Pyrenees by sequential sampling molar teeth for isotopic analysis. This analytical framework is expected to be useful to elucidate past environments and herding practices of pastoralist communities found at Cova de Els Trocs.

**References**

1. Balasse M, Ambrose SH. Mobilité altitudinale des pasteurs néolithiques dans la vallée du Rift (Kenya) : premiers indices de l'analyse du δ13C de l'émail dentaire du cheptel domestique. Anthropozoologica. 2005;40:147–66.

2. Tornero C, Balasse M, Bălăşescu A, Chataigner C, Gasparyan B, Montoya C. The altitudinal mobility of wild sheep at the Epigravettian site of Kalavan 1 (Lesser Caucasus, Armenia): evidence from a sequential isotopic analysis in tooth enamel. Journal of Human Evolution. 2016;97:27-36.

3. Tornero C, Aguilera M, Ferrio JP, Arcusa H, Moreno-García M, García-Reig S, et al. Vertical sheep mobility along the altitudinal gradient through stable isotope analyses in tooth molar bioapatite, meteoric water and pastures: A reference from the Ebro valley to the Central Pyrenees. Quaternary International. 2018;484 (10):94-106.

4. Makarewicz CA. Sequential δ13C and δ18O analyses of early Holocene bovid tooth enamel: resolving vertical transhumance in Neolithic domesticated sheep and goats. Palaeogeogr Palaeoclimatol Palaeoecol. 2017;485:16–29.

5. Makarewicz CA, Arbuckle BS, Öztan A. Vertical transhumance of sheep and goats identified by intra-tooth sequential carbon (δ13C) and oxygen (δ18O) isotopic analyses: Evidence from Chalcolithic Köşk Höyük, central Turkey. Journal of Archaeological Science. 2017;86:68-80.

6. Knockaert J, Balasse M, Rendu C, Burens A, Campmajo P, Carozza L, et al. Mountain adaptation of caprine herding in the eastern Pyrenees during the Bronze Age: a stable oxygen and carbon isotope analysis of teeth. Quaternary International. 2018;484:60–73.

7. Janzen A, Balasse M, Ambrose SH. Early pastoral mobility and seasonality in Kenya assessed through stable isotope analysis. Journal of Archaeological Science. 2020;117:105099.

8. de Bello F, Lepš J, Sebastià M-T. Predictive value of plants traits to grazing along a climatic gradient in the Mediterranean. Journal of Applied Ecology. 2005;42:824-33.

9. de Bello F, Buchmann N, Casals P, Lepš J, Sebastià M-T. Relating plant species and functional diversity to community δ13C in NE Spain patures. Agriculture, Ecosystems and Environment 2009;131:303-7.

10. Farquhar GD, Ehleringer JR, Hubick KT. Carbon Isotope Discrimination and Photosynthesis. Annual Review of Plant Physiology and Plant Molecular Biology. 1989;40:503-37.

11. Jumpponen A, Mulder CPH, Huss-Danell K, Hogber P. Winners and losers in herbaceous plant communities from foliar carbon isotope composition in monocultures and mixtures. Journal of Ecology. 2005;93:1136-47.

12. Caldeira MC, Ryel RJ, Lawton JH, Pereira JS. Mechanism of positive biodiversity-production relationships: insights provided by δC-13 analysis in experimental Mediterranean grassland plots. Ecology Letters. 2001;4,:439-43.

13. Peñuelas J, Filella I, Terradas J. Variability of plant nitrogen and water use in a 100-m transect of a subdesertic depression of the Ebro valley (Spain) characterized by leaf δ13C and δ15N. Acta Oecologica 1999;20:119-23.
